# Understanding adolescent girls’ experiences with accessing and using contraceptives in Zambia

**DOI:** 10.1186/s12889-023-17131-3

**Published:** 2023-11-03

**Authors:** Mumbi Chola, Khumbulani Hlongwana, Themba G. Ginindza

**Affiliations:** 1 Discipline of Public Health Medicine, School of Nursing and Public Health, University of KwaZulu-Natal, Durban, 4041 South Africa; 2 Department of Epidemiology and Biostatistics, School of Public Health, University of Zambia, Lusaka, 10101 Zambia; 3 Cancer & Infectious Diseases Epidemiology Research Unit (CIDERU), College of Health Sciences, University of KwaZulu-Natal, Durban, 4041 South Africa

**Keywords:** Adolescent girls, Contraceptive use, Experiences, Zambia

## Abstract

**Background:**

Globally, the unmet need for contraception among adolescent girls is high and is driven by barriers to access and utilisation of contraceptives. Understanding adolescent girls’ experiences with accessing and using contraceptives is crucial because it influences their decision to use and willingness to continue using health products and services. While determinants of contraceptive use have been extensively researched globally, few studies explore how adolescent girls experience contraceptive use in Zambia using qualitative methods. Therefore, this study aimed to understand Zambian adolescent girls’ experiences using contraceptives.

**Methods:**

Thematic analysis was used to analyse data generated from 7 focus group discussions and three in-depth interviews with adolescent girls aged 15 to 19 years in 4 districts in Zambia. NVivo version 12 pro (QSR International) software was used to manage and organise the data.

**Results:**

Results revealed that adolescents’ experiences concerning contraceptives across the continuum of care are shaped by various factors, including knowledge of contraceptives which comprises sources of information and contraceptives; experience with using contraceptives, challenges with access to contraceptives, and misconceptions about contraceptives; perspectives about existing contraceptives; and preferred types of contraceptives.

**Conclusion:**

The multifactorial interaction relating to adolescents’ personal experience, their community and the environment in which they access contraceptive services all contribute to their overall experience and influence their contraceptive decisions. Therefore, qualitative studies exploring adolescents’ experiences with accessing and using contraceptives are vital for tailoring interventions responsive to the contraceptive needs of this age group.

## Background

Globally, women of reproductive age, particularly adolescents, have a high unmet need for contraception. Estimates in 2017 showed that in developing regions, 214 million women aged 15–49 years had an unmet need for contraception [1]. Over the period 1970 and 2019, the lowest rates of demand satisfaction for contraceptives were among adolescents (15–19 years) and young adults (20–24 years), with 64.8% and 71.9%, respectively, with 43·2 million of them having an unmet need for contraception in 2019 [2]. Key reasons for this unmet need for contraception are attributed to various barriers to accessing and using contraceptives, such as limited access to and choice of contraceptive methods, fear of or experience of side effects, cultural or religious opposition, poor quality of available services, and gender-based barriers [1]. These are barriers based on the experiences that adolescents have as they access and use contraceptives. Their experience is determined by client-related and healthcare-related factors, which comprise (1) the client’s lived experience, (2) the client’s subjective influences, (3) the quality of healthcare services, (4) the responsiveness of the health system to non-health needs of clients, (5) and the politics of healthcare and the perspectives of healthcare providers [3]. The manifestation of the adolescent girls’ experience is the result of this experience and typically shows through client satisfaction and client engagement, with positive client experiences leading to increases in client satisfaction and client engagement [3].

Client satisfaction is based on the client or client’s experience after using a health service and shows how much they like/dislike the service after using it [4]. It is an element of the client’s overall experience and has been used to understand client experience with the healthcare system and ensure the quality of service [5]. Globally, it has been used as an essential indicator of service acceptability, clients’ decision to use, and willingness to continue using health products and services [6–8]. Studies have shown that provider-client communication, waiting times and staff compassion and empathy are influential determinants of client experience [9]. With regard to family planning, factors such as client-provider communication, the level of trust the client has and the type, confidentiality, health provider skills, and level of information and counselling that is provided to them positively influenced the client’s knowledge regarding contraceptives, the client’s satisfaction with the healthcare provider and the contraceptive method they choose, and the use of more effective contraceptive methods [4, 10].

The experience that adolescent girls have with accessing and using contraceptives influences their contraceptive decisions and may incline them towards not using contraceptives or discontinuing their use. Negative lived experiences with contraceptives among women are a core reason for discontinuation or change of contraceptive methods [11]. These negative experiences stem primarily from the side effects emanating from their contraceptive use [12–16]. Healthcare provider attitudes and behaviours also influence contraceptive decisions [17]. Health provider attitudes negatively impact the adolescent girls’ experience through their lack of competence, poor service delivery and unfriendly attitude, limited stock of contraceptives, and prohibitive costs [18, 19]. They hold misconceptions regarding contraceptives and usually have negative attitudes towards providing contraceptives to adolescents and young people, use unauthorised discretion to impose age restrictions which are not evidence-based, even request consent when it is not required [20, 21], and take a paternalistic attitude towards adolescents [16, 22, 23]. Positive staff attitudes have an impact on client satisfaction and improved client outcomes. Empathetic health providers who are keen on clients’ needs and preferences encourage family involvement and customise educational efforts to the individual, increase client satisfaction, adherence to recommended treatments, and client outcomes [24].

Stockouts, limited options for contraceptive methods, lack of privacy and confidentiality, and long waiting times [20] all shape adolescents’ perceptions of health facilities, which may affect their decision-making regarding contraceptive use [20]. These perceptions are formed based on the information that adolescents have about contraceptives. Adolescents often have inadequate, incomplete and incorrect information about contraceptives [21, 25, 26]. The types and sources of information they receive are often unreliable [12, 13, 21, 27, 28] but shape how they view the health services, products and the overall healthcare system.

While determinants of contraceptive use have been extensively researched globally and in the sub-region, few studies explore how adolescent girls experience contraceptive use, particularly in Zambia, employing qualitative methods. Therefore, this study sought to explore the experiences of Zambian adolescent girls with accessing and using contraceptives.

## Methods

### Study design

This study was conducted through a qualitative exploratory design that sought to understand adolescent girls’ experiences with accessing and using contraceptives in four districts in Zambia.

### Study setting

The study was done in Chongwe, Lusaka, Kasama and Luwingu districts located in Lusaka and Northern provinces, respectively. The two districts in each province were purposively selected based on whether they were predominantly rural or urban. Kasama and Lusaka districts in the Northern and Lusaka provinces are predominantly urban, while Chongwe (Lusaka province) and Luwingu (Northern province) are predominantly rural. In this study, one health facility with functional and active Youth Friendly Services was selected in each district with the help of the District Adolescent Health Coordinator. The Ministry of Health operates these corners through their health facilities, providing sexual and reproductive health IEC materials and products such as condoms and contraceptives to adolescents. Focus group discussions were held at these sites.

### Study participants and recruitment

Purposive sampling, specifically maximum variation sampling, was employed to recruit adolescent girls between 15 and 19 years to participate in the focus group discussions (FGDs) and Indepth interviews (IDIs). Peer educators from the selected health facilities assisted with the identification of potentially eligible adolescents and allowed the principal investigator to recruit participants based on the eligibility criteria. The criteria included only female participants who were residents of the study sites, aged between 15 and 19 years and accessed services from the youth-friendly spaces. Maximum variation sampling ensured that participants were diverse in age, knowledge of contraceptives, and educational levels.

### Data collection

A total of 7 FGDs, comprising 8–12 participants, and 3 IDIs were undertaken, each lasting between 60 and 90 min, following the signing of informed consent and assent forms by parents/ guardians or girls ≥ 18 years and girls below 18 years, respectively. The 3 IDIs at Mtendere clinic were conducted as opposed to an FGD because the participants were not comfortable with participating in an FGD but were willing to participate through IDIs. The FGDs and IDIs were all conducted by two trained and experienced research assistants under the supervision of the principal investigator. In addition to being experienced in qualitative research and fluent in local languages, the research assistants were taken through the study protocol, consent documents and data collection tools. The FGDs and IDIs were conducted using the participant’s language of choice, which included English, Bemba and Nyanja and audio recorded (with participants’ permission). Throughout the study processes, confidentiality was maintained. Table 1 provides details on the types of interviews conducted and the participants involved.

**Table 1:** Description of types of interviews conducted and participants involved

Type	Health Facility	District	Province	Number of participants	Age Range	Education level
FGD	Mtendere Clinic	Lusaka	Lusaka Province	12	15–19	Primary and Secondary School
IDIs	Mtendere Clinic	Lusaka		3	15–19	Primary and Secondary School
FGD	Chongwe Clinic	Chongwe		9	15–19	Primary and Secondary School
FGD	Chongwe Clinic	Chongwe		8	15–19	Primary and Secondary School
FGD	Chikoyi Clinic	Luwingu	Northern Province	10	15–19	Primary and Secondary School
FGD	Chikoyi Clinic	Luwingu		10		Primary and Secondary School
FGD	Location Urban Clinic	Kasama		10	15–19	Primary and Secondary School
FGD	Namalundu Clinic	Kasama		10	15–19	Primary and Secondary School
			**Total**	**72**		

### Data analysis

Thematic analysis was used as a method to identify, analyse, and report patterns or themes observed within data [29], was used in this study. Audio materials from the interviews and FGDs were transcribed, and the ones in local languages were translated verbatim into English by the research assistants. NVivo version 12 pro (QSR International) software was used to manage and organise the data. Through an iterative process, one master code list was developed by the researchers. The process involved constantly reviewing the codes and the transcripts throughout the analysis to establish connections within the themes. Through this process, emerging themes were reviewed and, where needed, merged with other similar themes until the main themes and sub-themes were developed, which formed the master code list. Participant verbatim quotes were also used to ensure the credibility of the findings.

## Results

Results from this study revealed that adolescents’ experiences concerning contraceptive use across the continuum of care is influenced by various factors such as, experience with knowledge of contraceptives, including sources of information about contraceptives; experience with contraceptives accessing contraceptives, including the various challenges that come with this; experience with using contraceptives including misconceptions about contraceptives; and given these experiences, their preferred types of contraceptives.

### Experience with information on contraceptives

#### Knowledge of contraceptives

Participants were aware of and understood what contraceptives were. When asked what they understood by contraceptives, they described contraceptives as methods of preventing pregnancy and a means of planning for children.

“When I just hear the word family planning, the first thing that comes to mind is a person wanting time to decide when to have a child.“ *[FGD 6, participant 3, Location Urban Clinic, Kasama]*.

“It’s a way of preventing unwanted pregnancies. You are not ready to get pregnant, but you want to have sex.“ *[FGD 1, participant 5, Location Urban Clinic, Kasama]*.

“Methods of preventing pregnancies as one waits for the right time to get pregnant.“ *[FGD 5, participant 4, Chikoyi Clinic, Luwingu]*.

Beyond just knowing what contraceptives were, participants were also knowledgeable and aware of the different methods, such as pills, IUDs, injectables and condoms, that they could use.

“We can use pills, female condoms, injectables.“ *[FGD 5, participant 4, Chikoyi Clinic, Luwingu]*.

“There is another one whose name I can’t remember……… Yes! Microgynon pill.“ *[FGD 1, participant 6, Location Urban Clinic, Kasama]*.

Regarding emergency contraceptive pills, one participant had this to say.

“If I have unprotected sex with my boyfriend, I need to take it immediately or within 72 hours.“ *[FGD 6, participant 7, Location Urban Clinic, Kasama]*.

#### Sources of information on contraceptives

The research participants had different sources from which they got their information on contraceptives. Some of the primary sources of information were health facilities, specifically clinics and youth-friendly spaces, friends, and through the media such as television, radio and the internet.

“Health facilities, we get information on Depo from mother to child health care. They explain to us how it works.“ *[FGD 1, participant 2, Chongwe Clinic, Chongwe]*.

“From the youth-friendly corner, I see a lot of people come to get contraceptives. They are usually sensitised and given information about the contraceptives before they choose which one they want.“ *[FGD 6, participant 1, Location Urban Clinic, Kasama]*.

“There are adolescent programs, for example, on Lutanda radio station or Mano, they do bring people to sensitise on contraceptives. For example, every Friday, there is a program where they speak about such.“ *[FGD 6, participant 7, Location Urban Clinic, Kasama]*.

“It was on the morning after pill. My friend told me that after having sex, you need to use it within three days after having sex.“ *[FGD 6, participant 2, Location Urban Clinic, Kasama]*.

Other sources of contraceptive information included parents, relatives, partners, non-governmental organisations (NGOs), and schools. However, with parents, information was provided mainly to adolescents who already had children to deter them from having more children.

“With parents, unless they see that you have exceeded having children [had a lot of children], that is when they will tell you to go for family planning.“ *[IDI 1, participant, Mtendere Clinic, Lusaka]*.

There were mixed sentiments about the contraceptive information provided in schools. Information provided through established curricula, such as comprehensive sexual education and subjects like science and some clubs, was largely viewed as positive, while teachers’ opinions on contraceptives were seen as judgemental.

“From school through JETS club. They teach about different topics [on contraceptives].“ [*FGD 2, participant 5, Chongwe Clinic, Chongwe]*.

“At school, from the teachers in subjects like science [they address contraceptives].“ *[FGD 5, participant 4, Chikoyi Clinic, Luwingu]*.

However, participants raised concerns about teachers’ judgemental and anti-sex attitudes towards the girls who use contraceptives. As one participant mentioned,

“In schools, teachers are so judgemental. Each time you ask about contraceptives, they start saying things like, “At your age, you have started having sex”. This makes pupils uncomfortable to approach teachers to get information on contraceptives. That is why most of them chose to go to the clinic.“ *[FGD 7, participant 2, Namalundu Clinic, Kasama]*.

Participants also raised concerns about the lack of a conducive environment in schools, hindering them from freely approaching teachers for information. Teachers’ inability to maintain confidentiality when adolescents sought information on contraceptives and often using this against them by discussing their sexual activity in class was another deterrent.

“At Kasama Secondary [School], what I observed is that they do not create that environment where pupils can freely approach teachers to talk about contraceptives. For a pupil to talk to a teacher, that conducive environment. You will find that if you ask about contraceptives from a teacher, that teacher will use that against you in class by saying, ‘Some of you in here have started having sex”.“ *[FGD 7, participant 2, Namalundu Clinic, Kasama]*.

Among all these different sources of information, the most trusted were the health facility and NGOs, such as DREAMS.

“For me, it’s DREAMS and clinic because they will not only give me [the contraceptives] but will explain to me more about the contraceptive and how it works and how to use it.“ *[FGD 7, participant 3, Namalundu Clinic, Kasama]*.

Some participants preferred health facilities because they had youth-friendly spaces where they can get information and contraceptives.

“Because we have youth-friendly corners where you can get them [contraceptives], and the people there are open with them [contraceptives].“ *[FGD 7, participant 2, Namalundu Clinic, Kasama]*.

### Experience with accessing contraceptives

#### Sources of contraceptives

With regard to sources of contraceptives, participants preferred health facilities, drug stores and pharmacies, community outreach staff, and NGOs, mainly due to ease of access.

“It’s very easy at the clinic as people there know what you will need and will also give you the information you need.” *[FGD 5, participant 3, Chikoyi Clinic, Luwingu]*.

“Certain contraceptives such as condoms are found in shops. One can easily go and buy, especially if the hospital or clinic is far.” *[FGD 5, participant 6, Chikoyi Clinic, Luwingu]*.

“The reason why I said drug store is because if you know the contraceptive you want and have used before, it is easy to walk in a drug store, buy it and use it.” *[FGD 7, participant 2, Namalundu Clinic, Kasama]*.

#### Most used contraceptives

While participants used various contraceptives, the most commonly used were injectables, implants, condoms, pills and emergency contraceptives. The most widely used injectables were *Depo-Provera [injectable with 13-week cycle]* and *Sayana Press [injectable with 13-week cycle]*, preferring the 3-month duration, while those who used implants used Jadelle.

“I used Sayana press for 3 months.” *[FGD 1, participant 9, Chongwe Clinic, Chongwe]*.

“I used Depo for 3 months.” *[FGD 1, participant 7, Chongwe Clinic, Chongwe]*.

Some participants opted to discontinue the use of injectables in favour of other types of contraceptives, such as emergency contraceptive pills, which are taken after engaging in unprotected sex.

“When I started using Depo, I did it for 3 times, 3 months each. But I stopped, and I started using morning-after pills after sex.” *[FGD 1, participant 2, Chongwe Clinic, Chongwe]*.

Some participants reported carrying both male and female condoms with them in case their partners did not have one.

“As a lady, you need to be ready just in case he does not come with a condom, you can give him. This is so that we prevent diseases and unwanted pregnancies.” *[FGD 5, participant 4, Chikoyi Clinic, Luwingu]*.

Some participants used *Microgynon [Levonorgestrel/Ethinylestradiol]* and *Mycrolut [Levonorgestrel 30 µg tablets]* oral pills and mentioned the failure of other contraceptives, such as condom burst, as the reasons for their choice.

“They [adolescent girls] do not trust condoms so much. They [condoms] feel like they may burst. For pills, they [adolescent girls] know it will work.” *[FGD 4, participant 2, Chikoyi Clinic, Luwingu]*.

#### Challenges with access to contraceptives

Participants reported various challenges with accessing contraceptives, including staff attitudes at health facilities, lack of privacy and confidentiality, availability of contraceptives, expired drugs, and lack of information on contraceptives, specifically how they work, how to use them and their side effects.

#### Health facility staff attitude

The bad staff attitudes towards adolescent girls were more pervasive among the older staff, as they tended to ask many unsettling questions.

“The facial expression and the mentality of them seeing the young people as though they do not listen, even when you are the first to line up, they tell you to wait and go behind as though we are babies.” *[FGD 3, participant, Mtendere Clinic, Lusaka]*.

“Some staff at the youth-friendly spaces, especially the old ones, tend to ask us questions like, “Where are you taking these contraceptives? You are still young to be taking them.” *[FGD 1, participant 6, Location Urban Clinic, Kasama]*.

#### Lack of confidentiality and privacy

Some participants reported that health facility staff’s knowledge of their parents compromised confidentiality. Adolescent girls feared that staff would tell their parents about their trying to get contraceptives from the health facility.

“Others you will find that some nurses start telling their parents that they wanted to access contraceptives from the clinic.” *[FGD 1, participant 4, Chongwe Clinic, Chongwe]*.

“They are not very comfortable because most of the workers here know our parents, so they might end up disclosing to our parents that we access the contraceptives. So, it is very uncomfortable.” *[FGD 1, participant 4, Chongwe Clinic, Chongwe]*.

The lack of privacy was also another challenge raised by the participants. The locations where they access contraceptives within the health facilities are open and visible to everyone.

“At the clinic, a challenge we find is that the place where we can access them is too open. Many people can see us. If it was private, it would be better.” *[FGD 5, participant 10, Chikoyi Clinic, Luwingu]*.

#### Expired Drugs

Participants also reported challenges with expired drugs, particularly emergency contraceptives procured from drug stores or pharmacies.

“I will talk about drug stores; for example, you had sex with your boyfriend, and you buy a morning after pill and drink, and it shows no sign that it is working… maybe it’s expired. So, it’s hard to trust buying morning after pill in a drug store” *[FGD 6, participant 4, Location Urban Clinic, Kasama]*.

“I once bought a pill, and I did not check the expiry date. Afterwards, I discovered that it was expired.” *[FGD 4, participant 3, Chikoyi Clinic, Luwingu]*.

#### Unavailability of contraceptives and lack of information

The non-availability of contraceptives at times was a challenge for participants, which they feared would lead to them falling pregnant. Participants were concerned that they sometimes do not find contraceptives when they need them.

“Sometimes you find that the contraceptives are not there, and this might make us pregnant.” *[FGD 1, participant 2, Chongwe Clinic, Chongwe]*.

“In most of these drug stores, you only find salespersons who do not know anything about these contraceptives. So, they do not give you full information on contraceptives.” *[FGD 7, participant 7, Namalundu Clinic, Kasama]*.

### Experiences with using contraceptive use

Participants had varying experiences regarding contraceptive use, depending on the contraceptive type. These experiences also included the side effects of using contraceptives.

#### Experience with condoms

Not enjoying sex with a condom, preferring “skin to skin”, and fears of the condom breaking were commonly reported experiences.

“A condom is not nice unless skin on skin.” *[FGD 1, participant 7, Chongwe Clinic, Chongwe]*.

“We do not enjoy it because sometimes we are scared that it might break due to friction.” *[FGD 1, participant 1, Chongwe Clinic, Chongwe]*.

Others reported reactions such as itching in the vagina or their partners reacting and having a rash on the penis, and one girl stated that it caused stomach pains.

“Some condoms are nice, but others say that the condoms react on them [condom users react to the condoms], and they end up having a rash on their penises.” *[FGD 1, participant 3, Chongwe Clinic, Chongwe]*.

“For me, condoms make the vagina itch.” *[FGD 7, participant 7, Location Urban Clinic, Kasama]*.

“Sometimes even stomach aches. I do not know if it is the flavour they put on the condom.” *[FGD 7, participant 3, Location Urban Clinic, Kasama]*.

Another participant blamed condoms for delaying climax for some men, and the girls get tired of having sex.

“It does not feel nice because some men do not release quickly, and I get tired easily.” *[FGD 1, participant 2, Chongwe Clinic, Chongwe]*.

#### Experience with injectable contraceptives

Concerning injectables, one participant tried different injectable contraceptives and had similar experiences with all of them, including prolonged and frequent menstrual cycles with one contraceptive, delayed start of menstrual cycles with another contraceptive, and irregular periods with yet another contraceptive. She also experienced similar effects with using contraceptive pills.

“Jadelle contraceptive for three years made me have prolonged and frequent menstrual cycles not until they removed it. I then changed to Depo, which gave me similar problems. I later switched to Noristerat injection, which gave me a small challenge where my menstrual cycle would begin two days after the end of a cycle. I took Sayana *[Injectable contraceptive]*, and it still gave me similar problems of having a period for two weeks, one week without, and then it starts again. Pills also gave me the same problem.” *[FGD 7, participant 6, Namalundu Clinic, Kasama]*.

#### Side effects

Participants associated contraceptives with various side effects, including diarrhoea, dizziness, headaches, numbness in the hand, prolonged periods, stomach pains and weight gain.

“I have headaches, and sometimes my menstruating days increase, and the bleeding is too much. I also feel dizzy when I am walking.” *[FGD 2, participant 2, Chongwe Clinic, Chongwe]*.

“It is just okay. It is just that the moment you get the shot, your hand will seem like it is paralysed for some time.” *[FGD 2, participant 6, Chongwe Clinic, Chongwe]*.

“It [injectable contraceptive] makes me gain weight.” *[IDI participant 3, Mtendere Clinic, Lusaka]*.

“I have headaches and stomach pains.” *[FGD 2, participant 5, Chongwe Clinic, Chongwe]*.

### Misconceptions about contraceptives

Misconceptions about contraceptives also form part of the experience of using contraceptives. Participants raised concerns about misconceptions regarding contraceptives, including contraceptives cause clots, infertility, and cancer, contraceptives should not be used by women who have never been pregnant before, and that they can dislodge and move to other parts of the body.

#### Causing clots

This was regarding contraceptive pills that will form a clot in the body.

“Others hear a lot of misconceptions on contraceptives. For example, if you take pills, they will form a clot in your stomach” *[FGD 7, participant 6, Namalundu Clinic, Kasama]*.

#### Causing infertility

Participants reported having heard that contraceptives, particularly injectables and pills, cause infertility, especially in women who had not had a baby before.

“I hear that [the] injections affect your ability to conceive when you have never had a baby before.” *[FGD 1, participant 4, Chongwe Clinic, Chongwe]*.

#### Causing Cancer

This misinformation related to cervical cancer, as participants heard that contraceptives accumulate in the body and thus cause cancer, possibly due to toxicity.

“It brings cervical cancer because the medicine does not dissolve in the body, and it just accumulates.” *[FGD 2, participant 2, Chongwe Clinic, Chongwe]*.

#### Contraceptive dislodges and moves around the body

This refers particularly to implants. According to what the participants heard, the implants come out of position, move around in the body, and may go to the heart.

“The one they put on the arm for five years is known that it sometimes goes to the heart when you overwork your arm.” *[FGD 1, participant 9, Chongwe Clinic, Chongwe]*.

“Others are scared of the Loop because they heard that if inserted, it will move to your heart or in your blood.” *[FGD 7, participant 5, Namalundu Clinic, Kasama]*.

### Contraceptives are not for women who have never been pregnant before

There is also the misconception that women who have never been pregnant before should not use contraceptives. If they do, they should use non-hormonal contraceptives such as condoms.

“They say we [those who have children] should involve in family planning, and then for those that haven’t been pregnant before, they say it’s bad to do to family planning if you have never been pregnant before.” *[IDI 1 participant, Mtendere Clinic, Lusaka]*.

“If they didn’t have a child, I would advise them to go for a condom. For those with children, injections will help them in child spacing.” *[IDI 3 participant, Mtendere Clinic, Lusaka]*.

“A loop is designed for those who have had children before. They are not for us young ladies.” *[FGD 7, participant 10, Namalundu Clinic, Kasama]*.

### Perspectives about existing contraceptives

The participants had a narrow view of available contraceptive choices.

“They are what is available, so there is nothing we can do but use them. “*[FGD 4, participant 9, Chikoyi Clinic, Luwingu]*.

Some participants felt that the existing contraceptives were what they needed and were reliable, while others felt that more options were needed beyond what was available.

“They are what we need, especially for me, because they are reliable. If I have unprotected sex, I can easily go and get a contraceptive with that assurance that it will work.” *[FGD 7, participant 2, Location Urban Clinic, Kasama]*.

“I would like to say these injections and condoms used are not the only ones that should be available. I feel there should be more options.” *[FGD 2, participant 4, Chongwe Clinic, Chongwe]*.

### Preferred types of contraceptives

Participants had varying preferences when asked about their envisaged new contraceptives, with some wanting it to be a pill, others an injection, with some preferring a liquid contraceptive. Only one participant preferred to have a patch.

“It should be like a pill, just being able to drink it with water.” *[IDI participant 3, Mtendere Clinic, Lusaka]*.

“I would want it to be an injectable instead of the insertable. Some mission schools will not admit a child with an insertable contraceptive like Jadelle. Once they check and find you with a contraceptive of such nature, they deny you admission to the school.” *[FGD 7, participant 6, Namalundu Clinic, Kasama]*.

“I would want to see a liquid one. Something that we can just drink and not the pill.” *[FGD 5, participant 2, Chikoyi Clinic, Luwingu]*.

“I am not really a fan of injections and the pills I can be forgetting. I think I would go for a patch, the one for sticking on the shoulder.” *[FGD 1, participant 5, Chongwe Clinic, Chongwe]*.

They also preferred contraceptives that can be taken less frequently with minimal side effects and a longer duration of effectiveness.

“The one that should not give us any sort of problems [such as] dizziness, stomach problems, headaches or having a popping Stomach. *[IDI participant 1, Mtendere Clinic, Lusaka]*

“It should be a pill for taking once only, and it should be lasting for three years in the body, and it should not have any side effects.” *FGD 1, participant 2, Chongwe Clinic, Chongwe]*

“I would love to see a new pill that we could take on a monthly basis and not the current ones where you have to take one pill per day.” *[FGD 5, participant 3, Chikoyi Clinic, Luwingu]*.

“It should be a liquid contraceptive. One that you can drink only once after, say, two weeks.” *[FGD 4, participant 3, Chikoyi Clinic, Luwingu]*.

The participant who preferred a patch, when asked about the duration of effectiveness, she responded.

“Maybe changing it once a week can do.” *[FGD 2, participant 4, Chongwe Clinic, Chongwe]*.

The participants also wanted contraceptives that could prevent both pregnancy and diseases

“It can either be a pill or an injection, but once you take it prevent pregnancy and diseases.” *[FGD 5, participant 10, Chikoyi Clinic, Luwingu]*.

“I would love to see a pill that prevents both pregnancy and sexually transmitted diseases.” *[FGD 5, participant 1, Chikoyi Clinic, Luwingu]*.

The participants also wanted contraceptives to be readily available and easily accessible.

“The contraceptives should be easily accessible, and not today they are available, and tomorrow you don’t find them.” *[FGD 1, participant 3, Chongwe Clinic, Chongwe]*.

## Discussion

Client experience in healthcare, which is shaped by the healthcare system’s culture and influenced by clients’ perceptions across the continuum of care, the workforce experience and the community experience, can arguably be understood within the context of broader intrapersonal, community and environmental factors.

### Personal experience

Regarding contraceptive use among adolescent girls, since their experience is the sum of all interactions across the continuum of care. Their experience arguably starts with the girls seeking information about contraceptives once they get curious or intend to use them.

In our study, adolescent girls had some knowledge about contraceptives, consistent with findings from other studies [30, 31]. Adolescents were able to explain what contraceptives were, with examples of the different types of contraceptives. However, a deeper understanding of the different types of contraceptives and how they work, particularly hormonal contraceptives, was lacking, and other studies had similar results [13, 22, 23]. Health facilities, friends, media (such as television, radio, and the internet), schools, parents, relatives, partners, and non-governmental organisations, were the primary sources of information, some of which have been found to provide unreliable information [27, 32, 33]. Adolescent girls’ interaction with their community and the healthcare environment exposes them to misinformation or incomplete information about contraceptives. Health facility staff may provide accurate information, but they are prejudiced against adolescent girls based on their ages [25] and tend to take a parental role when dealing with them.

Similarly, information from peers, relatives and partners may not be accurate [13, 21, 34], as it is usually based on hearsay. As such, these informational sources usually exacerbate misinformation about contraceptives. In terms of the adolescent girls’ experience, the incomplete, inaccurate and inconsistent information they have about contraceptives is likely to influence their decisions about contraceptives. It may influence their perceptions about contraceptives and thus influence their willingness to use contraceptives. Adolescent girls may be less inclined to use contraceptives based on the misinformation they receive. Misinformation may also impact the correct use of contraceptives, which can further expose adolescent girls to pregnancy and associated poor health outcomes. Receiving inadequate information from a health provider has been associated with poor compliance with contraceptive use [35].

Misinformation also contributed to negative attitudes towards contraceptives. Negative attitudes about condoms have been reported in other studies, including condoms having “little holes” that make them ineffective in preventing STIs/HIV or pregnancy, free condoms obtained from clinics being defective, and condoms causing illnesses such as cancer, rashes, sores and stomach pains [36–40]. This also affects adolescents’ contraceptive experience and ultimately influences their contraceptive decisions, contributing to the non-use or discontinuation of use.

Part of the experience of contraceptive use is the sourcing of contraceptives. In this study, adolescent girls mentioned health facilities (hospitals and clinics), drug stores and pharmacies, community outreach staff and non-governmental organisations as sources of contraceptives. Similar sources have been mentioned in other studies [13, 26]. Among these sources, the girls considered health facilities, drug stores, and NGOs their most trusted sources. This could be because health facilities remain the main point of access to health care and as such are the main source of contraceptives and information on contraceptives particularly in rural areas where alternative information and contraceptive sources may be limited. Therefore, despite challenges with the attitude of health facility staff, health facilities remain a key and essential source of information on contraceptives and contraceptives themselves.

Adolescent girls also used the contraceptives that they obtained from their preferred sources. Among the most used contraceptives were injectables, implants, condoms, pills and emergency contraceptives, as found in other studies [41–43]. However, issues of availability of contraceptives, long waiting times and compromised privacy and confidentiality at health facilities [20] may lead to a poor experience and thus negatively influence their likelihood of using contraceptives. These also potentially impacted the continuity of use of contraceptives as adolescents were likely to discontinue use or be forced to switch to other methods, which could alter their experience.

As part of their experience with contraceptives, adolescents in this study reported various side effects, including prolonged and frequent menstrual cycles with one contraceptive, delayed start of menstrual cycles with another contraceptive, and irregular periods with yet another contraceptive, particularly among those using injectables. In contrast, those using condoms reported itching in the vagina, stomach pains or their partners having a rash on the penis. Other studies have reported similar side effects from contraceptive use experienced by adolescent girls. These have included irregular menstrual cycle [12, 13, 21], sickness, including abdominal pain [21, 25, 27], changes in weight (either extreme weight gain or loss) [13, 26–28, 44], menstrual cycle and colour of blood, spotting, constant bleeding [26, 44], amongst others. The adolescent girls have reported mostly negative experiences from using contraceptives which in most cases demotivates them from using contraceptives. While positive experiences have been associated with adherence and improved client outcomes [45], these negative experiences can lead to poor adherence to contraceptive use, thus exposing adolescents to pregnancy and the associated negative health outcomes.

### Community Experience

Part of the client experience is through interaction with the community. The communities in which adolescent girls live also shape how they perceive and experience contraceptive use. Through their interaction with friends, family and other community members, adolescent girls receive varying information and advice about contraceptives, either based on the experience of those who have used them or what those who have not used contraceptives have heard from those who have used them. This tends to result in misconceptions about contraceptives, how they work and their effects. In this study, some of the misconceptions the adolescent girls reported having heard included causing clots, causing infertility, causing cancer, contraceptives dislodging and moving around the body, or contraceptives perceived as not for women who have never been pregnant before. The fear of the effects of contraception on infertility or future fertility among adolescent girls and community members was a common concern [12, 21, 25–28]. Myths about hormonal contraceptives causing infertility have been widely reported and significantly influence adolescent girls’ contraceptive use [27]. Societal and cultural norms that disapprove of contraceptive use are also a key influence on adolescent girls’ experience, particularly their decisions to use contraceptives [13]. Most adult women believe contraception is unacceptable for adolescent girls and believe it harms health and future childbearing in nulliparous girls [46].

### Institutional factors

The environment in which the health services are provided also has an impact on the client experience, particularly with accessing contraceptives. With regard to contraceptive use, health facilities and other locations where contraceptive services and products are provided affect client experience, both in terms of access and actual usage. Adolescent girls in this study reported various challenges with accessing contraceptives, mostly centred around health facilities. The challenges raised included health facility staff attitude, lack of confidentiality and privacy, expired drugs, lack of availability of contraceptives, and lack of information on contraceptives. Institutional and environmental factors relating to accessing contraceptives have been reported among adolescents in other countries [13, 21, 25]. Attitudes of health facility staff, particularly harsh treatment and scolding by nurses, have been reported as a challenge and negatively influenced decisions to use contraceptives among adolescent girls [21]. The lack of privacy and health facilities, lack of a conducive environment and policies that hinder access to contraceptives also have a bearing on the client experience and tend to negatively influence contraceptive use among adolescents [25].

### Perspectives about existing and future contraceptives

Based on their experience with contraceptives, adolescent girls from this study had different perspectives about the contraceptives currently available to them and also what sort of contraceptives they would prefer. Even with their side effects, some adolescents felt they had no choice but to use them as they were what was available. Some felt that they needed more options beyond what was available. Suggestions were made on what options they would like, which can be considered regarding delivery methods, effectiveness and side effects. Several delivery methods in addition to the ones already available (pills and injections) were suggested, such as liquid contraceptives and patches which can be attached to the skin. Concerning effectiveness, adolescents would like contraceptives that they can take less frequently and with a longer duration of effectiveness. They would also like contraceptives that have minimal side effects and can prevent pregnancies and diseases. Relating to access to contraceptives, they would like contraceptives to be easily and readily accessible.

### Study limitations

Study participants were recruited from youth-friendly services/corners, which may have biased recruitment to those inclined to seek health services from or live near the health centres. Therefore, applying these results to settings dissimilar to the study sites may not be possible. Future studies should consider different approaches when recruiting participants to enable the generalisability of study findings.

## Conclusion

Client experience is at the core of the human experience in healthcare and typically starts when the client first interacts with the healthcare system, continues through the continuum of care and is not a once-off experience but can happen multiple times. This study has highlighted the experience that adolescent girls have concerning accessing and using contraceptives. The interaction of factors related to their personal experience, their community and the environment in which they access contraceptive services all contribute to the overall client experience and ultimately influence the adolescent girls’ decision to use contraceptives. Therefore, any intervention targeting improving contraceptive use among adolescent girls should be cognisant of these complexities. Non-use and discontinuation of use is typically the case when the experience is negative, and most adolescent girls have a negative client experience when accessing and using contraceptives due to challenges such as negative attitudes of healthcare staff, problems with accessing contraceptives, and lack of confidentiality, as well as the side effects from the contraceptives themselves. This ultimately inclines them not to use contraceptives. Therefore, ensuring that adolescent girls have a positive client experience when they access and use contraceptives is vital in programme and health facility efforts to improve contraceptive use in this age group.

## Data Availability

Data can be accessed upon request through the corresponding author.

## Abbreviations

AOR: Adjusted Odds Ratio
CI: Confidence Interval
CPH: Census of Population and Housing
DHS: Demographic and Health Survey
LICs: Low-Income Countries
UKZNBREC: University of KwaZulu Natal Biomedical Research Ethics Committee
WHO: World Health Organization
ZDHS: Zambia Demographic and Health Survey
